# Supplementary material for: Ecogenomics of virophages and their giant virus hosts assessed through time series metagenomics
Source: Nat Commun. 2017 Oct 11;8:858. doi: 10.1038/s41467-017-01086-2 (PMC5636890; doi:10.1038/s41467-017-01086-2)
Supplement: Supplementary file 1 — Supplementary Information [file 41467_2017_1086_MOESM1_ESM.pdf]

Supplementary Figures and Tables

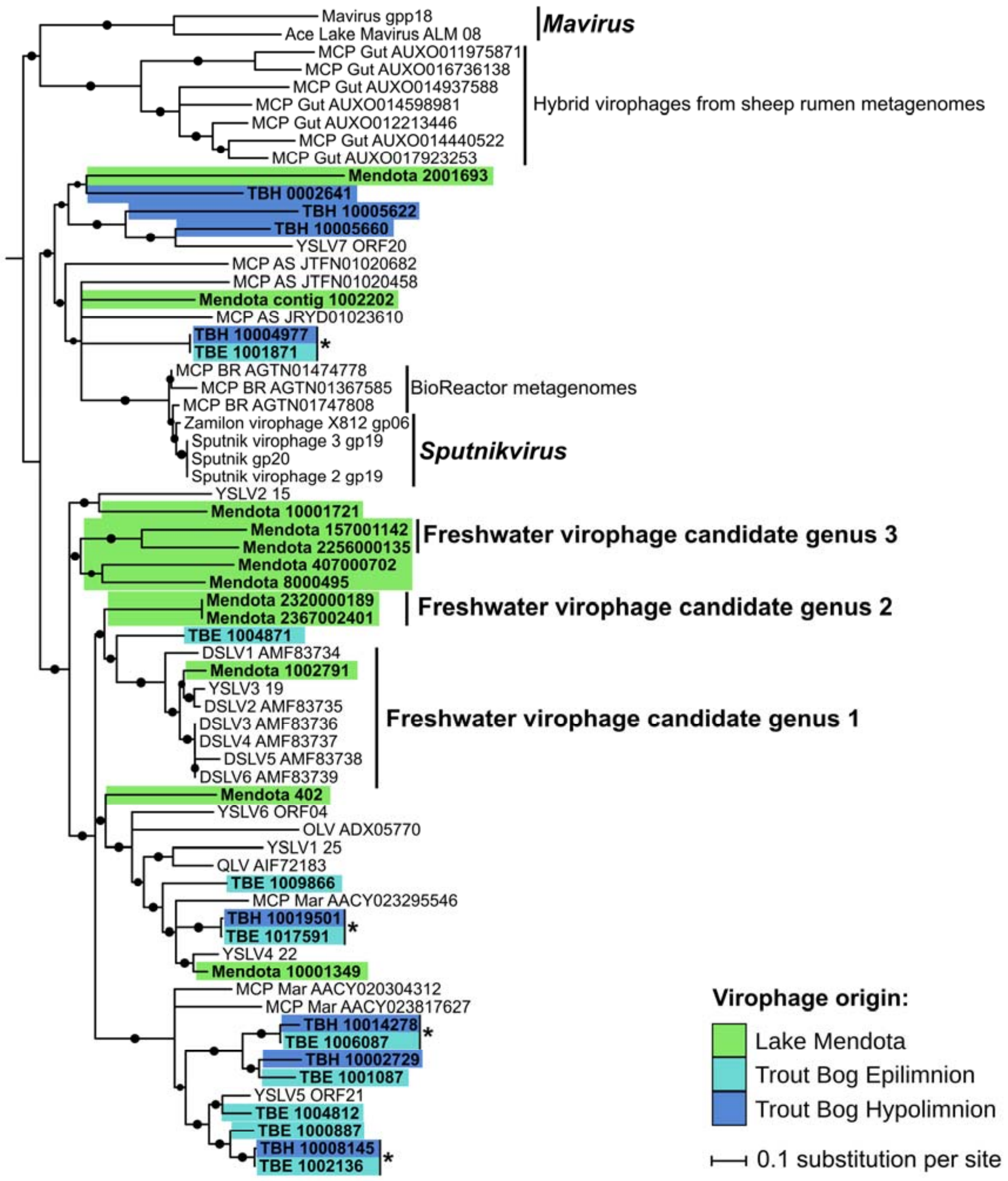

Supplementary Figure 1. Maximum-likelihood tree from virophage Major Capsid Protein (MCP). Branches with support < 50% are displayed as multifurcations. TBH: Trout Bog Hypolimnion, TBE: Trout Bog Epilimnion, YSLV: Yellowstone Lake virophage, OLV: Organic Lake virophage, QLV: Qinghai Lake virophage, DSLV: Dishui Lake virophage, ALM: Ace Lake Mavirus, Mar: Marine, AS: Activated Sludge, Gut: Sheep Rumen, BR: Bioreactor. MCP corresponding to identical virophage genomes assembled in both Trout Bog Epi- and Hypolimnion are highlighted with a star.

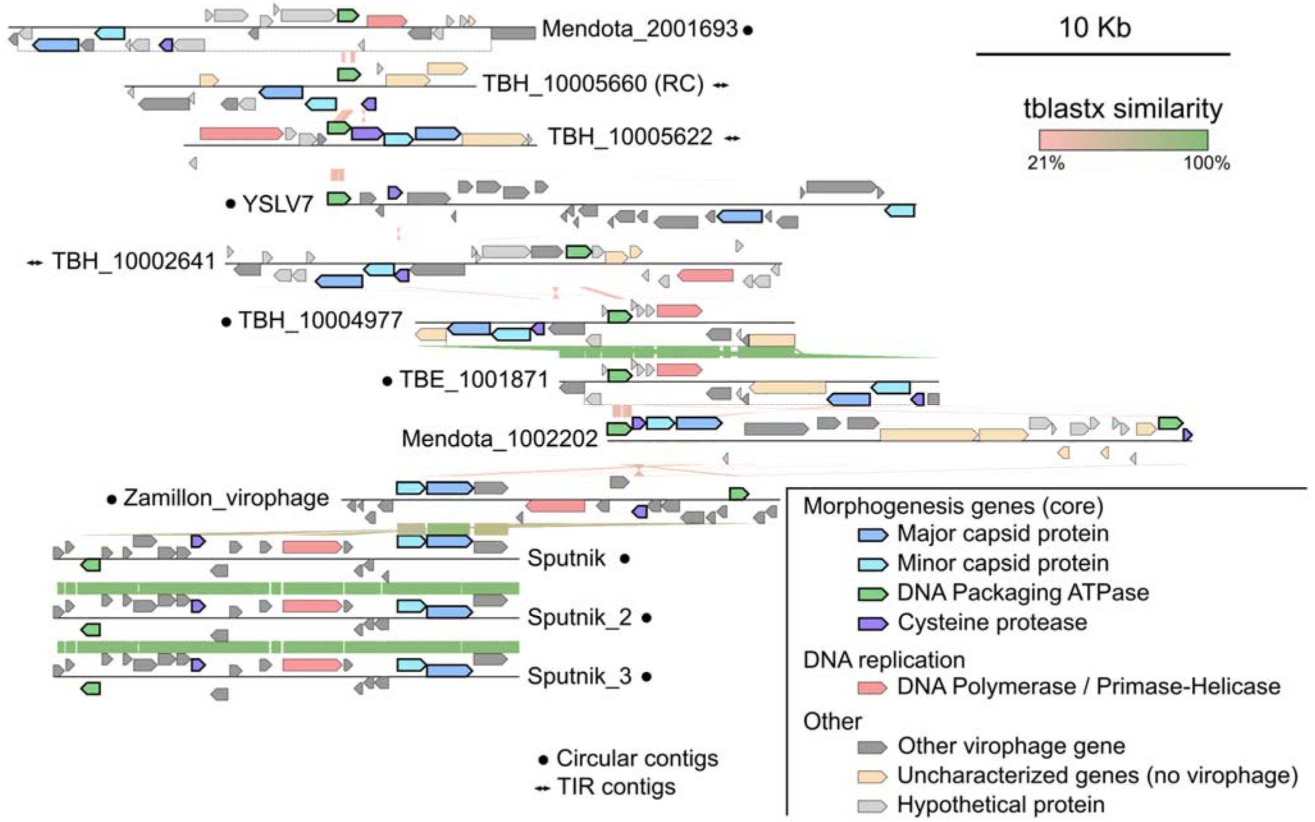

Supplementary Figure 2. Genome map comparisons for *Sputnikvirus* virophages and related uncultivated freshwater virophages (based on the MCP phylogeny, Fig. S1). The four core genes coding for the major capsid protein, minor capsid protein, ATPase (V3), and protease (V9) are highlighted in bold. The remaining genome comparisons are in Supplementary Fig. 3.

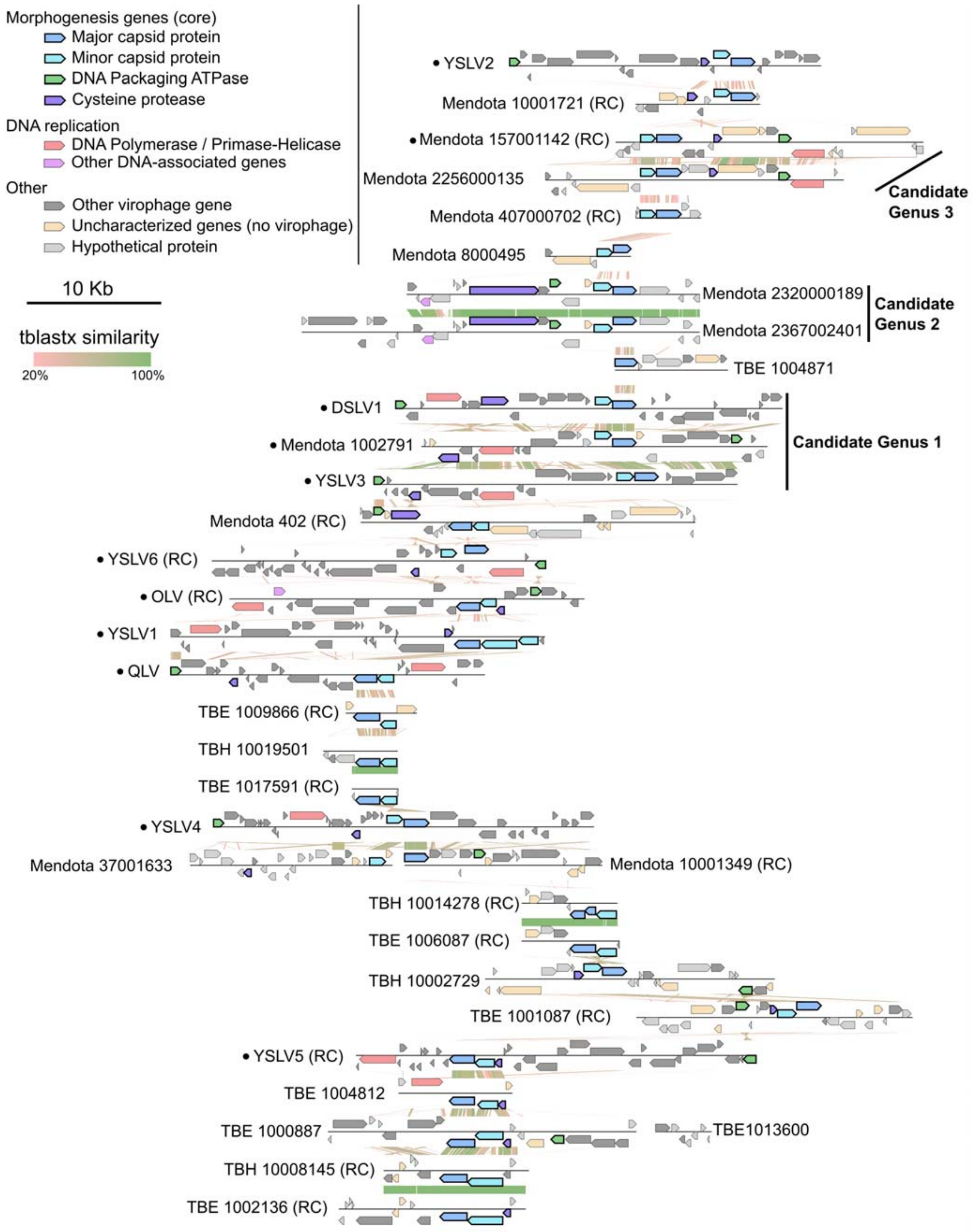

Supplementary Figure 3. Genome map comparisons for uncultivated freshwater virophages (remaining genome comparisons are available in Supplementary Fig 2). The four core genes coding for the major capsid protein, minor capsid protein, ATPase (V3), and protease (V9) are highlighted in bold.

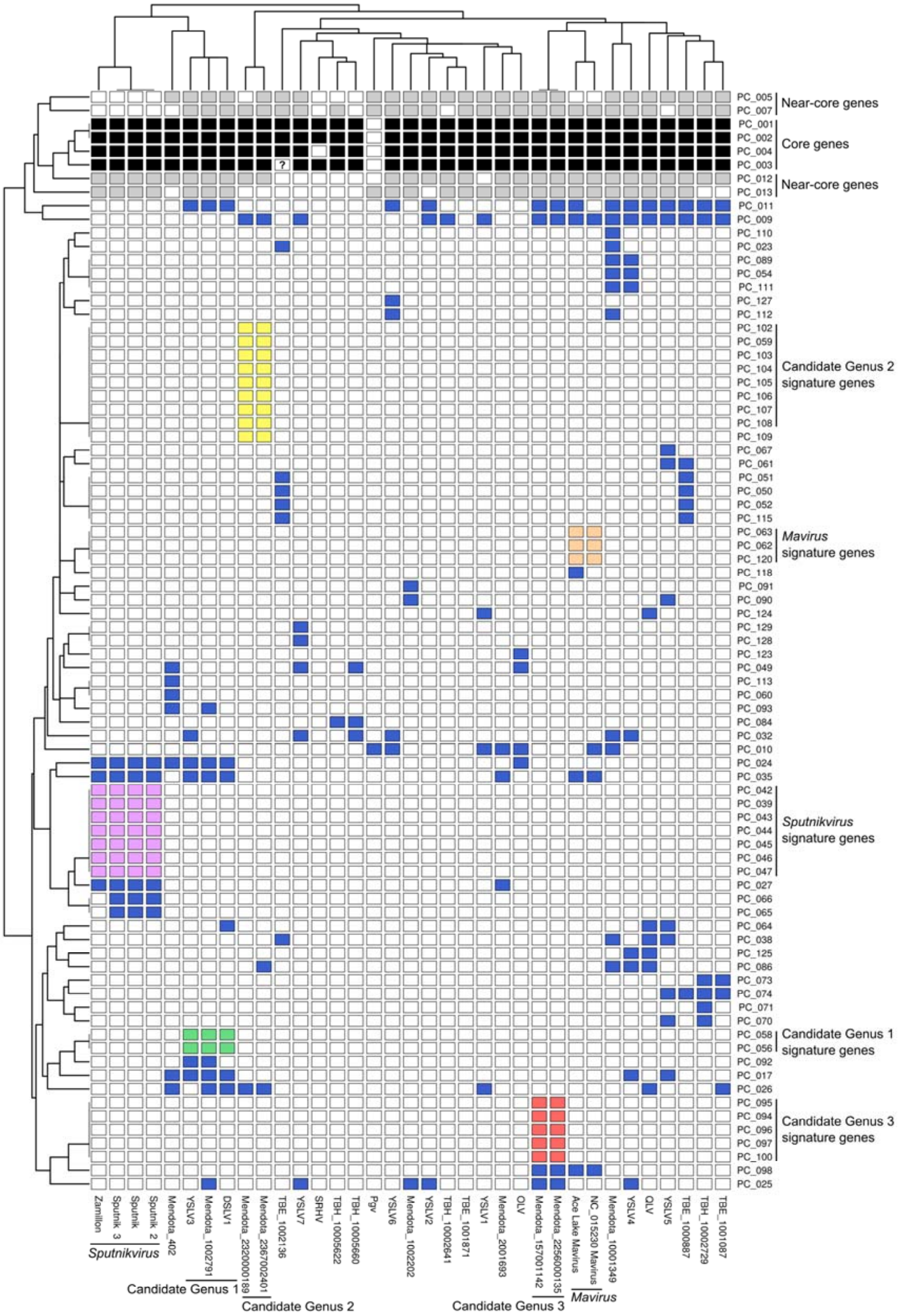

Supplementary Figure 4. Virophage genomes gene content heatmap. The heatmap depicts the presence of Protein Clusters (PCs, including at least two predicted proteins) across virophage complete and near-complete genomes (i.e. linear contigs > 13kb). PCs are colored as in Fig. 1.

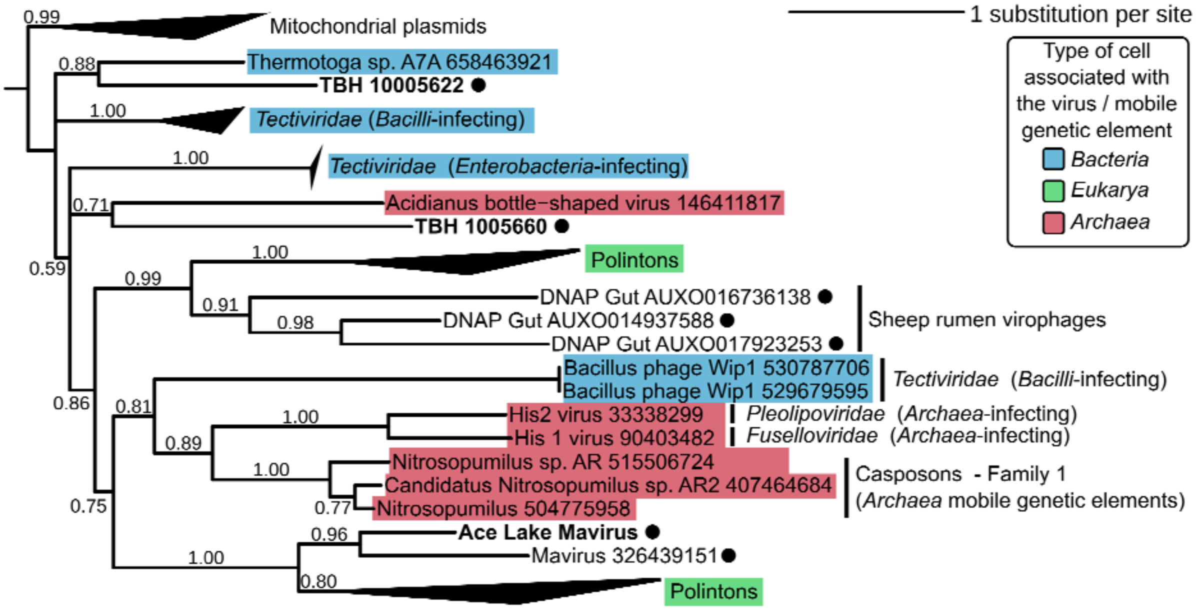

Supplementary Figure 5. Maximum-likelihood tree computed from virophage PolB sequences and related references. All branches with bootstrap < 0.5 were collapsed. PolB genes from virophages are highlighted with a black circle, and freshwater virophages are highlighted in bold. Other references are colored according to the affiliation of their cellular genome.

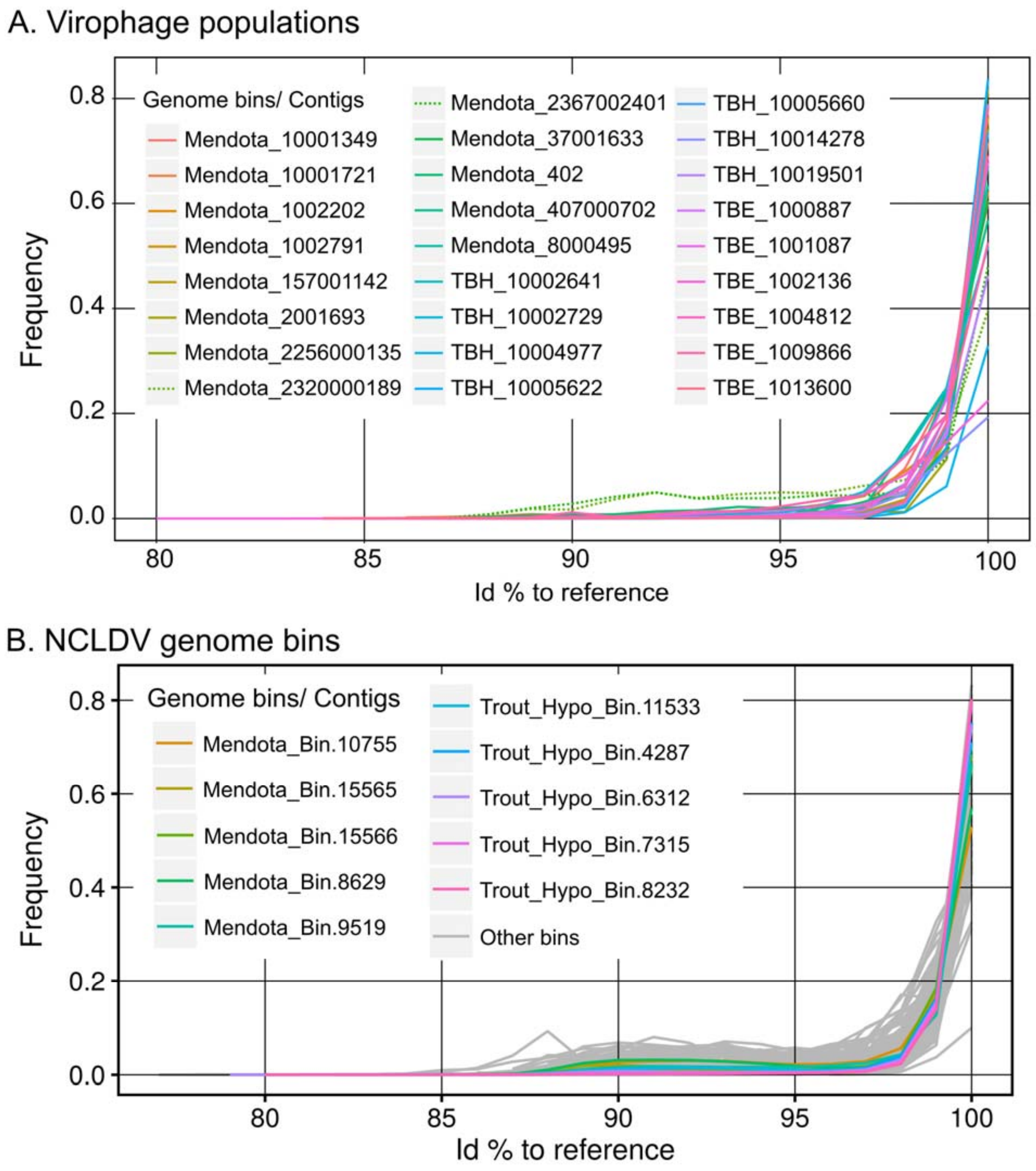

Supplementary Figure 6. Distribution of identity percentage for reads mapping to the assembled virophage (A) and NCLDV (B) genome bins. (A) The two overlapping virophage contigs with an unusual set of reads mapped at ~90% identity are displayed with dashed lines. (B) For clarity, only the 10 largest NCLDV bins are colored, the other being all displayed with gray lines.

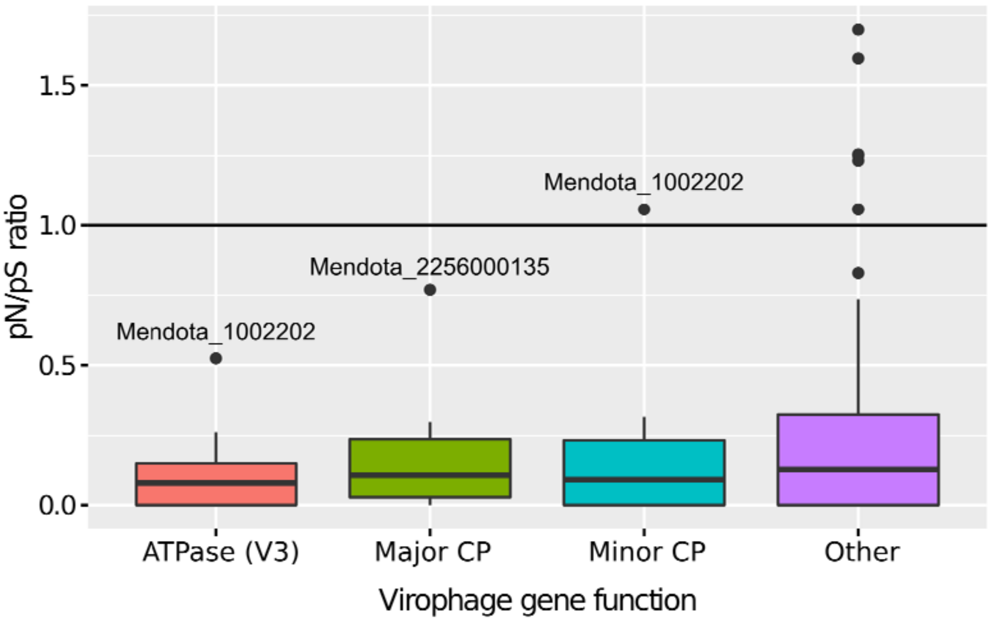

Supplementary Figure 7. Distribution of pN/pS ratios observed on virophage genes. The contig IDs of genes with an available functional affiliation and pN/pS value > 0.5 are indicated on the plot. Boxplots are constructed with the upper and lower lines corresponding to the 25th and 75th percentiles, and outliers displayed as points.

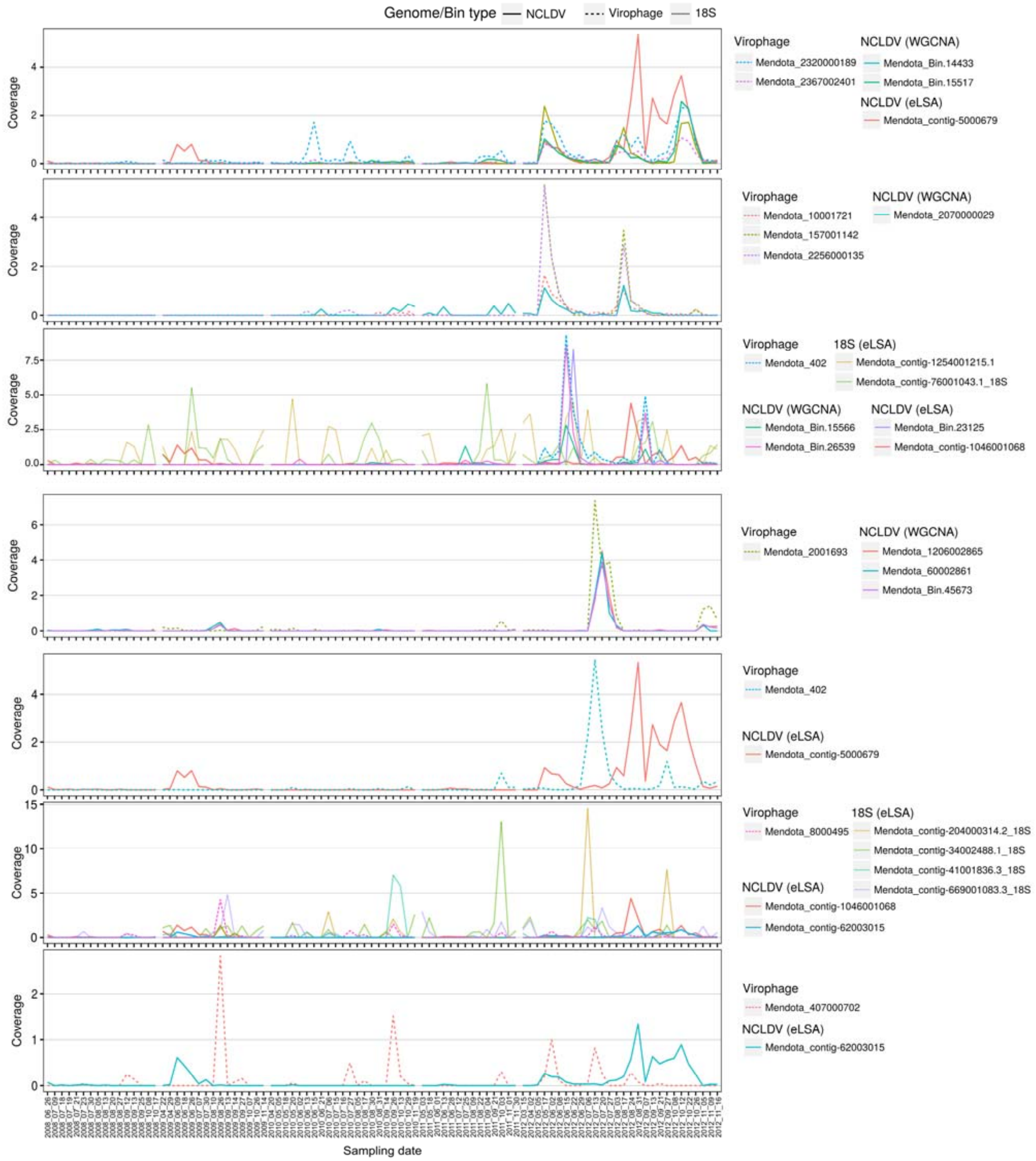

Supplementary Figure 8. Relative abundance of virophage and NCLDV contigs grouped by co-occurring modules (detected through WGCNA clustering) in Lake Mendota. The abundance profiles are presented only for modules including both virophage and NCLDV contigs.

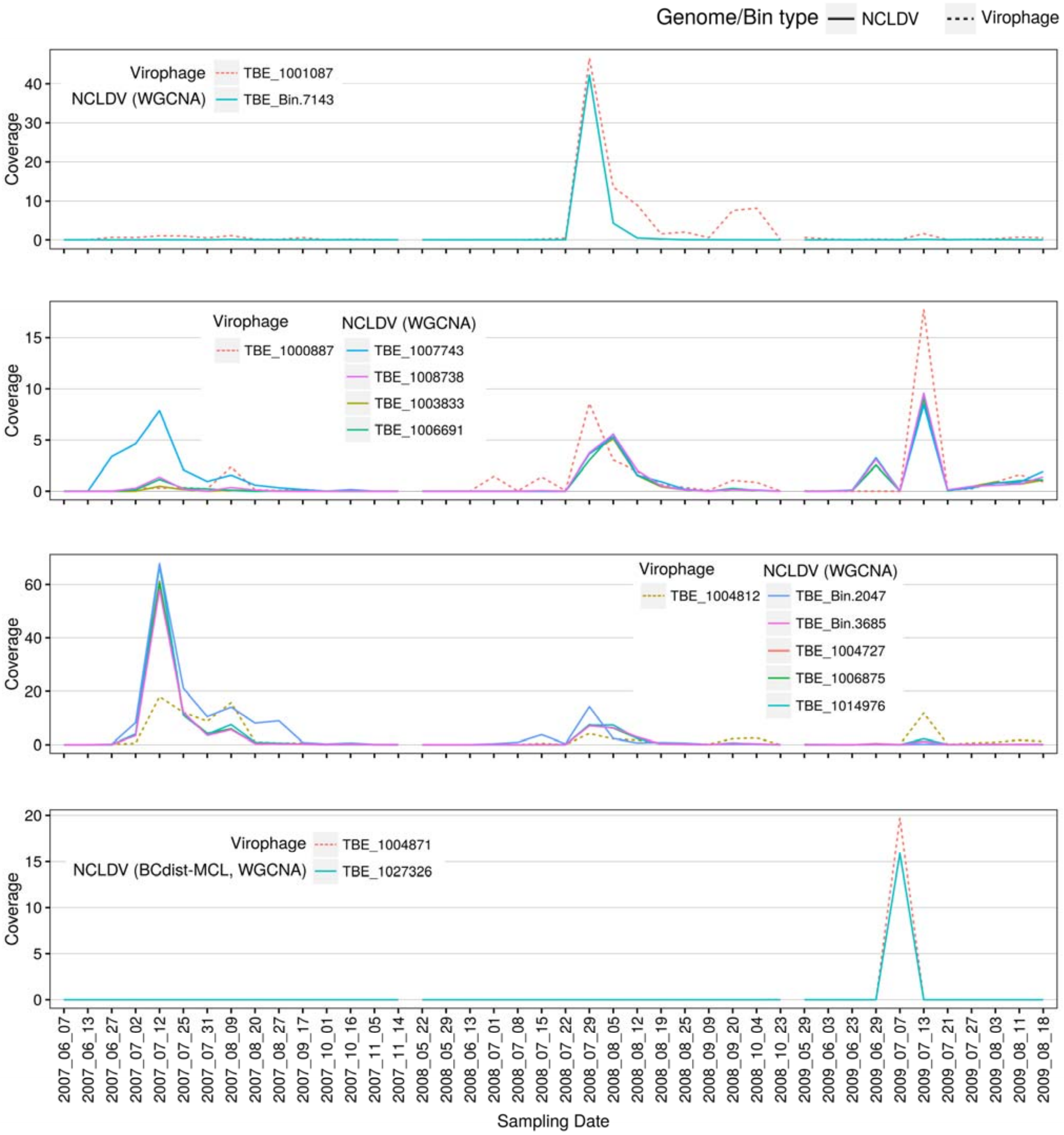

Supplementary Figure 9. Relative abundance of virophage and NCLDV contigs grouped by co-occurring modules (detected through WGCNA clustering) in Trout Bog Lake Epilimnion. The abundance profiles are presented only for modules including both virophage and NCLDV contigs.

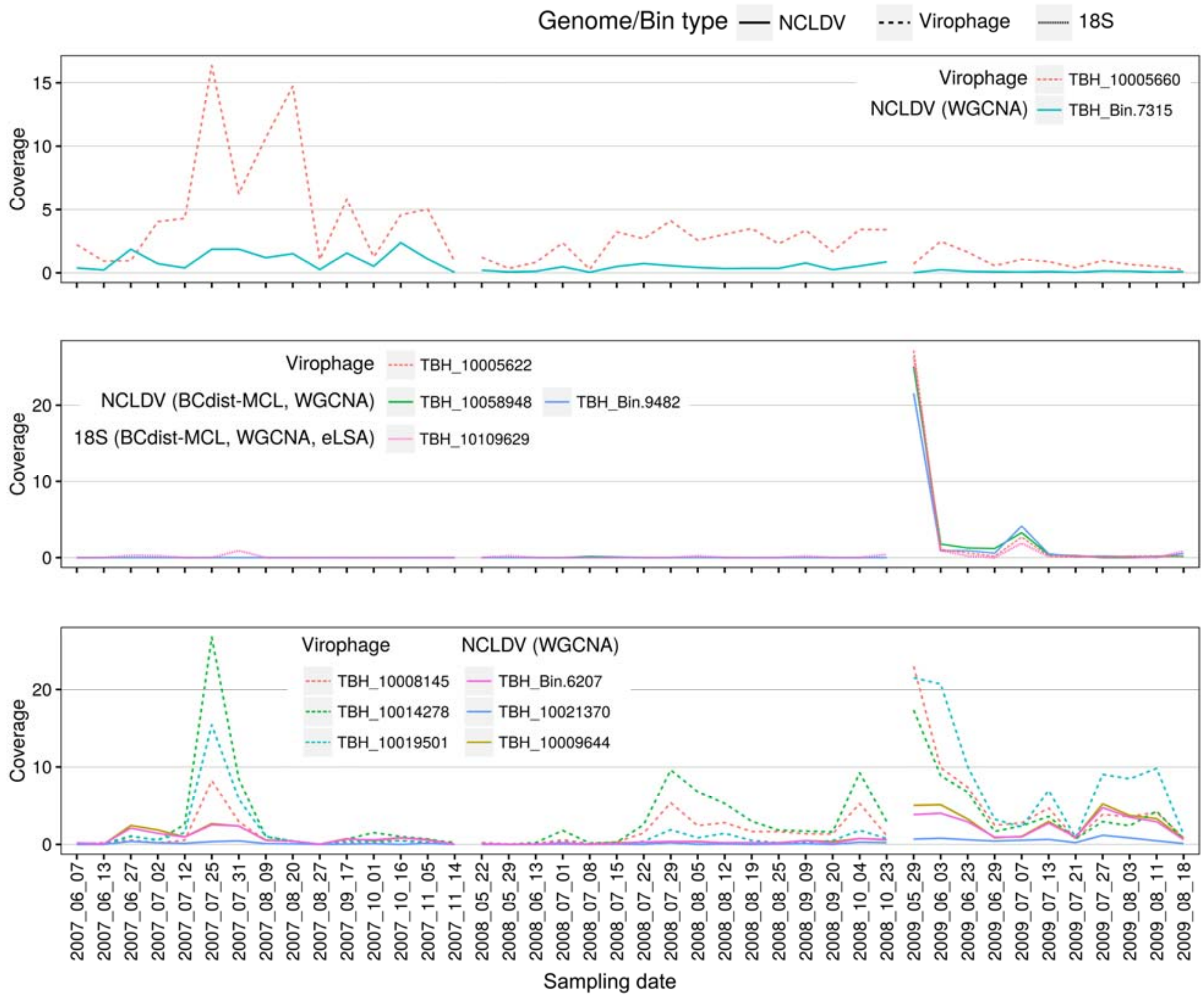

Supplementary Figure 10. Relative abundance of virophage, NCLDV, and 18S contigs grouped by co-occurring modules (detected through WGCNA clustering) in Trout Bog Lake Hypolimnion. The abundance profiles are presented only for modules including both virophage and NCLDV or 18S contigs.

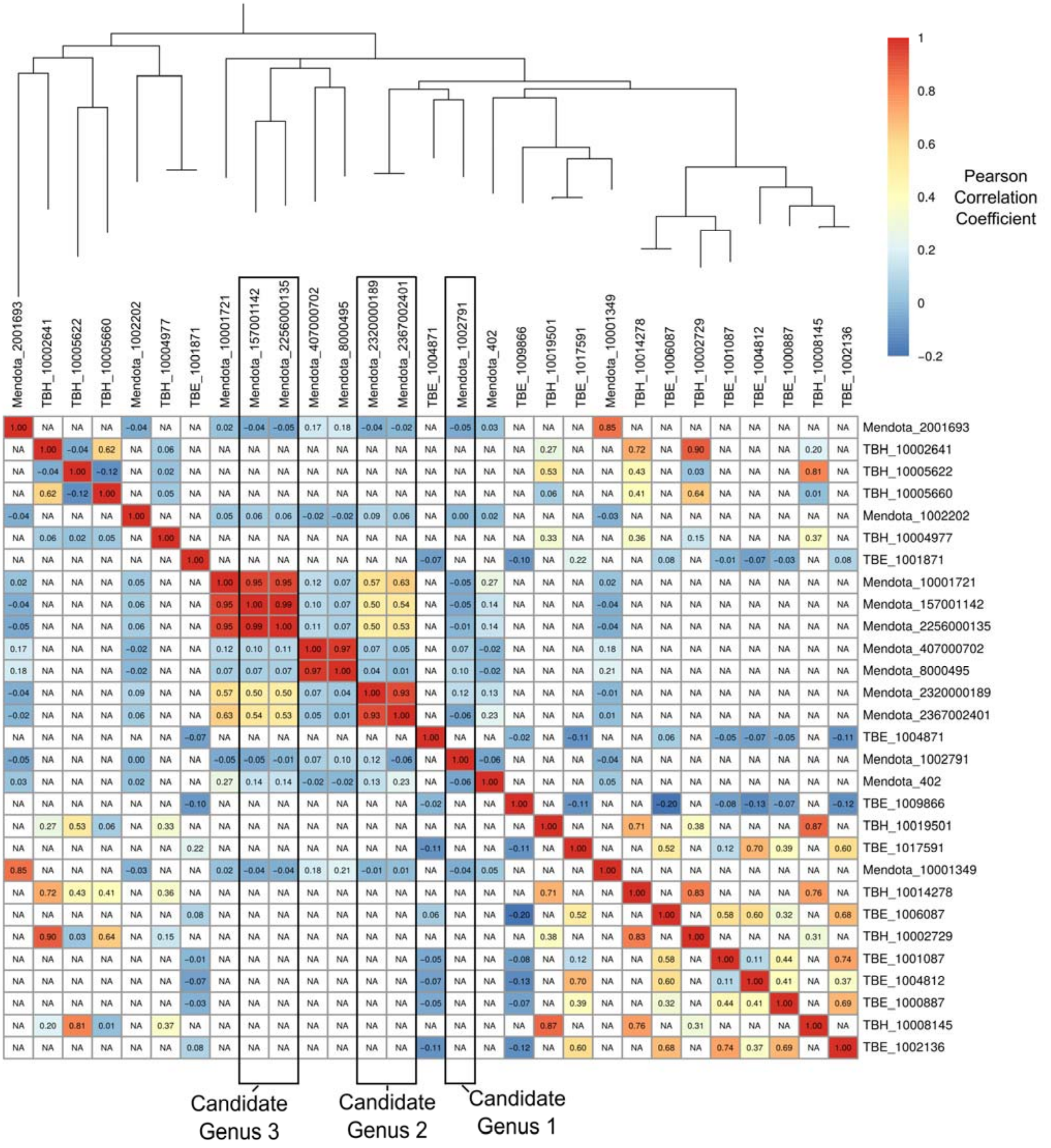

Supplementary Figure 11. Pearson correlations between abundance profiles of virophages from Lake Mendota, Trout Bog Epilimnion and Trout Bog Hypolimnion. Virophages were ordered based on the MCP phylogeny (Supplementary Fig. 1). Pearson correlations were computed only within each dataset (Lake Mendota, Trout Bog Lake Epilimnion, and Trout Bog Lake Hypolimnion).

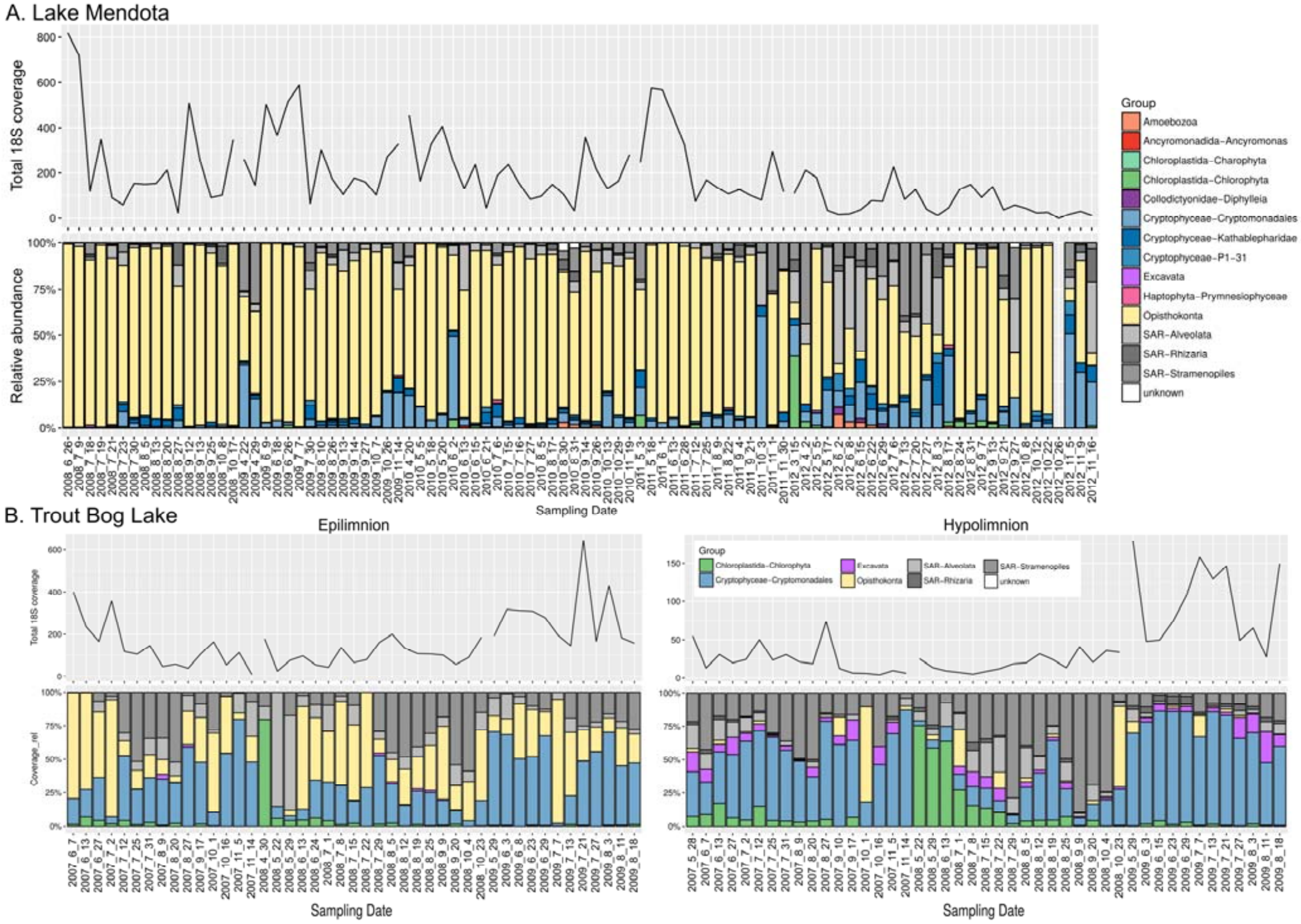

Supplementary Figure 12. Eukaryotic diversity (based on 18S sequences) in lake Mendota (A) and Trout Bog Lake Epi- and Hypolimnion (B). 18S sequences were affiliated based on a best BLAST hit against the SILVA database. No 18S sequences were detected for Lake Mendota Epilimnion sampled on 2012-10-26.

Lake / Water layer	Contig Id	Bin/Genome size	Circular (o) or TIR (x)	Additional contig in bin	# of predicted genes	# of ORFans	Identical contigs across different samples	Taxonomic classification (within the *Lavidaviridae* family)	WGCNA module
Lake Mendota / Epilimnion	Mendota_10001349	29 768		Mendota_37001633 (Mendota_Bin.29857)	36	21		unclassified	-
Lake Mendota / Epilimnion	Mendota_2367002401	29 610			24	5		Candidate genus 2	Mendota_turquoise
Lake Mendota / Epilimnion	Mendota_1002791	25 756	o		23	11		Candidate genus 1	-
Lake Mendota / Epilimnion	Mendota_402	24 917			23	10		unclassified	Mendota_yellow
Lake Mendota / Epilimnion	Mendota_1002202	23 012			22	10		unclassified	Mendota_mediumorchid
Lake Mendota / Epilimnion	Mendota_157001142	22 900	o		21	5		Candidate genus 3	Mendota_blue
Lake Mendota / Epilimnion	Mendota_2256000135	22 196			18	3		Candidate genus 3	Mendota_blue
Lake Mendota / Epilimnion	Mendota_2320000189	21 805			18	1		Candidate genus 2	Mendota_turquoise
Lake Mendota / Epilimnion	Mendota_2001693	20 751	o		23	13		unclassified	Mendota_darkgreen
Lake Mendota / Epilimnion	Mendota_10001721	9 285			10	5		unclassified	Mendota_blue
Lake Mendota / Epilimnion	Mendota_8000495	6 363			5	2		unclassified	-
Lake Mendota / Epilimnion	Mendota_407000702	4 860			6	4		unclassified	-
Trout Bog Lake / Epilimnion	TBE_1000887	27 128		TBE_1013600 (Trout_Epi_Bin.8427)	25	11		unclassified	TBE_grey60
Trout Bog Lake / Epilimnion	TBE_1001087	20 549			23	7		unclassified	TBE_blue
Trout Bog Lake / Epilimnion	TBE_1001871	14 947	o		15	8	++++	unclassified	-
Trout Bog Lake / Epilimnion	TBE_1002136	13 909			13	4	****	unclassified	-
Trout Bog Lake / Epilimnion	TBE_1004812	8 475			6	2		unclassified	TBE_tan
Trout Bog Lake / Epilimnion	TBE_1004871	8 404			7	5		unclassified	TBE_pink
Trout Bog Lake / Epilimnion	TBE_1006087	7 275			7	1	——	unclassified	-
Trout Bog Lake / Epilimnion	TBE_1009866	5 268			4	0		unclassified	-
Trout Bog Lake / Epilimnion	TBE_1017591	3 443			4	2	°°°°	unclassified	-
Trout Bog Lake / Hypolimnion	TBH_10002641	21 931	x		25	17		unclassified	TBH_yellowgreen
Trout Bog Lake / Hypolimnion	TBH_10002729	21 538			24	10		unclassified	TBH_yellow
Trout Bog Lake / Hypolimnion	TBH_10004977	14 947	o		15	8	++++	unclassified	TBH_orange
Trout Bog Lake / Hypolimnion	TBH_10005622	13 905	x		22	16		unclassified	TBH_green
Trout Bog Lake / Hypolimnion	TBH_10005660	13 842	x		13	6		unclassified	TBH_black
Trout Bog Lake / Hypolimnion	TBH_10008145	10 805			10	1	****	unclassified	TBH_violet
Trout Bog Lake / Hypolimnion	TBH_10014278	7 121			8	2	——	unclassified	TBH_violet
Trout Bog Lake / Hypolimnion	TBH_10019501	5 552			5	1	°°°°	unclassified	TBH_violet

Supplementary Table 1. Characteristics of virophage contigs identified in Lake Mendota and Trout Bog Lake metagenomes. TIR: Terminal Inverted Repeats (>100 nt). Virophage types are based on the 4-core genes phylogeny (Fig. 1).

Genome	Length (bp)	Total Coverage (x)	SNP density (SNP/kb)	Percentage of reads mapped at ≥95% id.	Percentage of reads mapped at ≥99% id.
Mendota_10001349	29 768	16.10	4.47	98.29%	84.41%
Mendota_2367002401	29 610	25.20	2.36	77.37%	59.53%
Mendota_1002791	25 756	75.94	3.96	98.75%	92.68%
Mendota_402	24 917	30.47	8.59	96.88%	84.68%
Mendota_1002202	23 012	43.80	11.91	94.91%	77.43%
Mendota_157001142	22 900	14.25	1.14	98.56%	93.27%
Mendota_2256000135	22 196	13.76	2.30	98.62%	89.24%
Mendota_2320000189	21 805	22.58	2.25	78.23%	54.88%
Mendota_2001693	20 751	22.78	0.87	97.78%	93.34%
TBE_1000887	27 128	51.10	0.00	99.70%	95.40%
TBE_1001087	20 549	144.14	3.11	99.52%	90.56%
TBE_1002136	13 909	233.03	17.04	92.76%	63.66%
TBH_10002641	21 931	79.71	0.73	97.82%	86.92%
TBH_10002729	21 538	97.73	11.70	99.63%	96.33%
TBH_10004977	14 947	122.24	0.13	97.36%	76.39%
TBH_10005622	13 905	68.25	0.58	99.60%	95.22%
TBH_10005660	13 842	182.59	0.36	98.56%	93.58%

Supplementary Table 2. Genetic diversity of (near-)completely assembled virophage populations. For each genome ≥ 13kb, the cumulated coverage across the whole dataset is indicated alongside the SNP density and percentage of reads mapping to the contig(s) at ≥ 95% and ≥ 99%. The two genomes for which < 90% of the reads map at ≥ 95% identity are highlighted in red.

Sample	Epi-2011_8_22	Epi-2011_9_4
1627_ref	9	2
1627_alt	3	
1629_ref	9	2
1629_alt	3	
1728_ref	12	1
1728_alt	5	1
1792_ref	15	4
1792_alt	5	1
1822_ref	15	3
1822_alt	4	1
1848_ref	15	3
1848_alt	5	2
1869_ref	16	3
1869_alt	6	2
1897_ref	15	4
1897_alt	5	2
1959_ref	18	5
1959_alt	4	3
1980_ref	20	5
1980_alt	4	2
2115_ref	22	3
2115_alt	5	2
2181_ref	4	3
2181_alt	22	4
2182_ref	4	3
2182_alt	22	4
2296_ref	16	5
2296_alt	5	3
2325_ref	17	5
2325_alt	4	2

Supplementary Table 3. Coverage of non-synonymous SNPs from Mendota_1002202 minor capsid protein. SNP coverage is presented for the two samples with Mendota_1002202 contig covered $\geq$ 4x (in columns). Each SNP is identified by its position in the alignment, and alleles are noted by ‘ref’ and ‘alt’.
