# Supplementary material for: Ecogenomics of virophages and their giant virus hosts assessed through time series metagenomics
Source: Nat Commun. 2017 Oct 11;8:858. doi: 10.1038/s41467-017-01086-2 (PMC5636890; doi:10.1038/s41467-017-01086-2)
Supplement: Supplementary file 3 — Description of Additional Supplementary Files [file 41467_2017_1086_MOESM3_ESM.pdf]

Description of Additional Supplementary Files

File Name: Supplementary Data 1
Description: List of core, near-core, and signature genes.

File Name: Supplementary Data 2
Description: Amino-acid identity (AAI) between virophage MCPs.

File Name: Supplementary Data 3
Description: Characteristics of freshwater NCLDV genome bins from Lake Mendota and Trout Bog Lake.

File Name: Supplementary Data 4
Description: Virophage hosts (NCLDV or 18S contigs) predicted from co-occurrence analysis.

File Name: Supplementary Data 5
Description: Eukaryote plankton diversity for Lake Mendota and Trout Bog Lake.

File Name: Supplementary Data 6
Description: List of metagenomic datasets from Lake Mendota and Trout Bog Lake.
